# Supplementary material for: Neuronal Menin Overexpression Rescues Learning and Memory Phenotype in CA1-Specific α7 nAChRs KD Mice
Source: Cells. 2021 Nov 24;10(12):3286. doi: 10.3390/cells10123286 (PMC8699470; doi:10.3390/cells10123286)
Supplement: Supplementary file 1 [file cells-10-03286-s001.zip › cells-1461955-supplementary.pdf]

Supplementary Table S1. SP8 Leica (confocal) microscope settings. (Hotchkiss Brain Institute Advanced Microscopy Platform: **HBIAMP**, University of Calgary, AB, Canada

**Fluorescent microscope settings for data presented in:**	**SP8 Leica LIA - Laser Wavelength**	**Laser Power**
Figure 2	405.7	4.5
	488.0	3.2
	580.0	5.0
Figure 3	402.7	5.0
	488.0	2.8
	580.0	4.5
Figure 4	405.7	3.5
	488	4.8
	580	3.6
	680.0	5.5
Figure 5	405.7	3.5
	488	4.6
	580.0	4.3
	680.0	4.5
Figure 6	405.7	2.5
	580.0	4.5
	488.0	5.7
Figure 7	402.7	2.5
	488.0	4.3
	580.0	5.5
Figure 8 A	402.7	2.5
	488.0	3.3
	680.0	6.5
Figure 8B	488.0	4.5
	580.0	5.0
	680.0	2.5
Figure 9	488.7	4.5
	580.0	5.3
	633.0	4.5
Figure 10	488.0	4.5
	580.0	4.0
	633.0	4.5
Figure 10	488.0	4.5
	580.0	4.0
	633.0	4.5

.

Supplementary Table S2. Related to Figure 1C. Statistical analysis of qPCR analysis of Multiple endocrine neoplasia type 1, *MEN1* gene, nAChRs neuronal subunits α2-7 and β2-4 on DIV10 in-vitro and DIV20

Tukey's multiple comparisons test	Mean Diff.	95.00% CI of diff.	Below threshold?	Summary	Adjusted P Value
sca7a7 vs. sha7a7	1.191	0.7825 to 1.600	Yes	****	<0.0001
sca7men vs. sha7men	-2.693	-3.102 to -2.284	Yes	****	<0.0001
sca7a5 vs. sha7a5	-1.223	-1.632 to -0.8143	Yes	****	<0.0001
sca7a2 vs. sha7a2	-0.06474	-0.4737 to 0.3443	No	ns	>0.9999
sca7a3 vs. sha7a3	0.07709	-0.3319 to 0.4861	No	ns	>0.9999
sca7a4 vs. sha7a4	0.05592	-0.3531 to 0.4649	No	ns	>0.9999
sca7a6 vs. sha7a6	0.05886	-0.3501 to 0.4679	No	ns	>0.9999
sca7b2 vs. sha7b2	-0.007246	-0.4162 to 0.4017	No	ns	>0.9999
sca7b3 vs. sha7b3	-0.09094	-0.4999 to 0.3181	No	ns	>0.9999
scab4 vs. sha7b4	-0.1199	-0.5289 to 0.2891	No	ns	0.9999
**DIV 20**					
sca7a7 vs. sha7a7	1.703	1.473 to 1.934	Yes	****	<0.0001
sca7men vs. sha7men	1.686	1.456 to 1.917	Yes	****	<0.0001
sca7a5 vs. sha7a5	-0.1975	-0.4280 to 0.03297	No	ns	0.1846
sca7a2 vs. sha7a2	-0.03451	-0.2650 to 0.1960	No	ns	>0.9999
sca7a3 vs. sha7a3	-0.08483	-0.3153 to 0.1456	No	ns	0.9976
sca7a4 vs. sha7a4	-0.04571	-0.2762 to 0.1848	No	ns	>0.9999
sca7a6 vs. sha7a6	0.1033	-0.1272 to 0.3337	No	ns	0.9783
sca7b2 vs. sha7b2	0.05525	-0.1752 to 0.2857	No	ns	>0.9999
sca7b3 vs. sha7b3	-0.07651	-0.3070 to 0.1540	No	ns	0.9994

scab4 vs. sha7b4	0.01810	-0.2124 to 0.2486	No	ns	>0.9999

One-way ANOVA followed by Post-hoc-Tukey’s multiple comparison test
qPCR (n=6, 3 independent experiments each, triplicate replicates)

Supplementary Table S3. Related to Figure 2B. Statistical analysis of ICC fluorescence of bungarotoxin labelled α7 nAChRs in hippocampal neuronal cultures DIV 3-20

Source of Variation	% of total variation	P value	P value summary	Significant?
Interaction	22.95	<0.0001	****	Yes
Row Factor	6.308	<0.0001	****	Yes
Column Factor	69.75	<0.0001	****	Yes

**Tukey's multiple comparisons test**	**Mean Diff.**	**95.00% CI of diff.**	**Below threshold?**	**Summary**	**Adjusted P Value**
DIV3: Control vs. α7 scrambled AAV	-0.3333	-5.478 to 4.811	No	ns	>0.9999
DIV3: Control vs. α7 shRNA AAV	-1.833	-6.978 to 3.311	No	ns	0.9962
DIV7: Control vs α7 scrambled AAV	-0.5833	-5.728 to 4.561	No	ns	>0.9999
DIV7: Control vs. α7 shRNA AAV	55.00	49.86 to 60.14	Yes	****	<0.0001
DIV10: Control vs. α7 scrambled AAV	-0.8333	-5.978 to 4.311	No	ns	>0.9999
DIV10: Control vs.α7 shRNA AAV	66.17	61.02 to 71.31	Yes	****	<0.0001
DIV14: Control vs. α7 scrambled AAV	-1.750	-6.894 to 3.394	No	ns	0.9977
DIV14: Control vs.α7 shRNA AAV	95.83	90.69 to 101.0	Yes	****	<0.0001
DIV 20: Control vs. α7 scrambled AAV	-1.167	-6.311 to 3.978	No	ns	>0.9999

DIV 20: Control vs. α7 shRNA AAV	94.08	88.94 to 99.23	Yes	****	<0.0001

One-way ANOVA followed by Post-hoc-Tukey's multiple comparison test
n refers to the analysis of bungarotoxin labelled α7 nAChRs puncta fluorescence intensity within neuronal cultures, ROIs derived from 30 separate images from 5 independent experiments, 6 samples

Supplementary Table S4. Related to Figure 3B. Statistical analysis of ICC fluorescence of menin-C in hippocampal neuronal cultures DIV 3-20

Two-way RM ANOVA	Matching: Both factors				
Assume sphericity?	No				
Alpha	0.05				
Source of Variation	% of total variation	P value	P value summary	Significant?	Geisser-Greenhouse's epsilon
Row Factor	48.69	<0.0001	****	Yes	0.6034
Column Factor	5.939	<0.0001	****	Yes	0.7392
Row Factor x Column Factor	43.06	<0.0001	****	Yes	0.4046
Subject x Row Factor	0.6239				
Subject x Column Factor	0.4324				
Subject	0.1858				

**Tukey's multiple comparisons test**	**Mean Diff.**	**95.00% CI of diff.**	**Below threshold?**	**Summary**	**Adjusted P Value**
DIV3:Control vs. DIV3:α7 scrambled AAV	-1.250	-5.704 to 3.204	No	ns	0.9917
DIV3:Control vs. DIV3:α7 shRNA AAV	-9.667	-13.47 to -5.863	Yes	****	<0.0001
DIV7:Control vs. DIV7:α7 scrambled AAV	1.333	-6.520 to 9.187	No	ns	>0.9999
DIV7:Control vs. DIV7:α7 shRNA AAV	-24.50	-31.00 to -18.00	Yes	****	<0.0001
DIV10:Control vs. DIV10:α7 scrambled AAV	-1.000	-4.253 to 2.253	No	ns	0.9827

DIV10:Control vs. DIV10:α7 shRNA AAV	-32.25	-36.70 to -27.80	Yes	****	<0.0001
DIV14:Control vs. DIV14:α7 scrambled AAV	-0.8333	-3.678 to 2.012	No	ns	0.9882
DIV14:Control vs. DIV14:α7 shRNA AAV	-19.42	-21.53 to -17.30	Yes	****	<0.0001
DIV 20:Control vs. DIV 20:α7 scrambled AAV	-1.000	-4.253 to 2.253	No	ns	0.9827
DIV 20:Control vs. DIV 20:α7 shRNA AAV	38.00	33.11 to 42.89	Yes	****	<0.0001

One-way ANOVA followed by Post-hoc-Tukey's multiple comparison test
n refers to the analysis of menin-C puncta fluorescence intensity within neuronal cultures, ROIs derived from 30 separate images from 5 independent experiments, 6 samples

Supplementary Table S5. Related to Figure 4B. Statistical analysis of ICC fluorescence of PSD-95 in hippocampal neuronal cultures DIV 3-20

**Tukey's multiple comparisons test**	**Mean Diff.**	**95.00% CI of diff.**	**Below threshold?**	**Summary**	**Adjusted P Value**
DIV3:Control vs. DIV3:α7 scrambled AAV	0.6667	-2.510 to 3.843	No	ns	>0.9999
DIV3:Control vs. DIV3:α7 shRNA AAV	-0.9167	-4.093 to 2.260	No	ns	0.9982
DIV7:Control vs. DIV7:α7 scrambled AAV	-1.583	-4.760 to 1.593	No	ns	0.8832
DIV7:Control vs. DIV7:α7 shRNA AAV	32.83	29.66 to 36.01	Yes	****	<0.0001
DIV10:Control vs. DIV10:α7 scrambled AAV	-2.750	-5.926 to 0.4262	No	ns	0.1601
DIV10:Control vs. DIV10:α7 shRNA AAV	37.33	34.16 to 40.51	Yes	****	<0.0001

DIV14:Control vs. DIV14:α7 scrambled AAV	1.750	-1.426 to 4.926	No	ns	0.7966
DIV14:Control vs. DIV14:α7 shRNA AAV	24.25	21.07 to 27.43	Yes	****	<0.0001

One-way ANOVA followed by Post-hoc-Tukey's multiple comparison test
n refers to the analysis of PSD-95 puncta fluorescence intensity within neuronal cultures, ROIs derived from 30 separate images from 5 independent experiments, 6 samples

Supplementary Table S6. Related to Figure 5B. Statistical analysis of ICC fluorescence of SYT in hippocampal neuronal cultures DIV 3-20

**Tukey's multiple comparisons test**	**Mean Diff.**	**95.00% CI of diff.**	**Below threshold?**	**Summary**	**Adjusted P Value**
DIV3:Control vs. DIV3:α7 scrambled AAV	-1.449	-5.814 to 2.917	No	ns	0.9940
DIV3:Control vs. DIV3:α7 shRNA AAV	-4.782	-9.148 to -0.4163	Yes	*	0.0190
DIV7:Control vs. DIV7:α7 scrambled AAV	0.000	-4.452 to 4.452	No	ns	>0.9999
DIV7:Control vs. DIV7:α7 shRNA AAV	-28.33	-32.79 to -23.88	Yes	****	<0.0001
DIV10:Control vs. DIV10:α7 scrambled AAV	0.5000	-3.952 to 4.952	No	ns	>0.9999
DIV10:Control vs. DIV10:α7 shRNA AAV	-33.92	-38.37 to -29.46	Yes	****	<0.0001
DIV14:Control vs. DIV14:α7 scrambled AAV	-2.917	-7.369 to 1.535	No	ns	0.5672
DIV14:Control vs. DIV14:α7 shRNA AAV	-25.92	-30.37 to -21.46	Yes	****	<0.0001

One-way ANOVA followed by Post-hoc-Tukey's multiple comparison test
n refers to the analysis of PSD-95 puncta fluorescence intensity within neuronal cultures, ROIs derived from 30 separate images from 5 independent experiments, 6 samples

Supplementary Table S7. Related to Figure 6B. Statistical analysis of qPCR analysis of Multiple endocrine neoplasia type 1, *MEN1* gene, nAChRs neuronal subunits α2-7 and β2-4 in CA1 hippocampal tissue of α7 knockdown (KD) mice versus its scrambled control

Tukey's multiple comparisons test	Mean Diff.	95.00% CI of diff.	Below threshold?	Summary	Adjusted P Value
sca7a7 vs. sha7a7	1.118	0.8411 to 1.395	Yes	****	<0.0001
sca7men vs. sha7men	1.114	0.8375 to 1.391	Yes	****	<0.0001
sca7a5 vs. sha7a5	0.2991	0.02234 to 0.5759	Yes	*	0.0202
sca7a2 vs. sha7a2	-0.2821	-0.5589 to -0.005361	Yes	*	0.0405
sca7a3 vs. sha7a3	-0.3566	-0.6334 to -0.07986	Yes	**	0.0014
sca7a4 vs. sha7a4	0.2134	-0.06335 to 0.4902	No	ns	0.3752
sca7a6 vs. sha7a6	-0.1404	-0.4171 to 0.1364	No	ns	0.9568
sca7b2 vs. sha7b2	-0.1176	-0.3944 to 0.1592	No	ns	0.9942
scaSYP vs. sha7SYP	-1.294	-1.570 to -1.017	Yes	****	<0.0001
scaPSD-95 vs. sha7PSD-95	0.6932	0.4164 to 0.9700	Yes	****	<0.0001
sca7b3 vs. sha7b3	-0.1110	-0.3878 to 0.1658	No	ns	0.9973
scab4 vs. sha7b4	-0.1194	-0.3962 to 0.1573	No	ns	0.9930

One-way ANOVA followed by Post-hoc-Tukey's multiple comparison test
qPCR (n=6, 4 independent experiments each, triplicate replicates)

Supplementary Table S8. Related to Figure 6C. Statistical analysis of IHC fluorescence of bungarotoxin labelled α7 nAChRs in hippocampal brain slices in 8 weeks old mice

Tukey's multiple comparisons test	Mean Diff.	95.00% CI of diff.	Below threshold?	Summary	Adjusted P Value
un-treated control vs. α7 scrambled AAV	-4.667	-13.91 to 4.580	No	ns	0.4395
un-treated control vs. α7 shRNA AAV	122.3	113.1 to 131.6	Yes	****	<0.0001
α7 scrambled AAV vs. α7 shRNA AAV	127.0	117.8 to 136.2	Yes	****	<0.0001

One-way ANOVA followed by Post-hoc-Tukey's multiple comparison test
n refers to the analysis of bungarotoxin labelled α7 nAChRs puncta fluorescence intensity within hippocampal brain slices, ROIs derived from 20 separate images from 5 independent experiments, 6 samples

Supplementary Table S9. Related to Figure 7B. Statistical analysis of IHC fluorescence of menin-C puncta in scrambled control and α7-KD hippocampal brain slices in 8 weeks old mice

**Tukey's multiple comparisons test**	**Mean Diff.**	**95.00% CI of diff.**	**Below threshold?**	**Summary**	**Adjusted P Value**
un-treated control vs. α7 scrambled AAV	-0.4167	-2.919 to 2.085	No	ns	0.9123
un-treated control vs. α7 shRNA AAV	94.42	91.91 to 96.92	Yes	****	<0.0001
α7 scrambled AAV vs. α7 shRNA AAV	94.83	92.33 to 97.34	Yes	****	<0.0001

One-way ANOVA followed by Post-hoc-Tukey's multiple comparison test
n refers to the analysis of menin-C puncta fluorescence intensity within hippocampal brain slices, ROIs derived from 20 separate images from 5 independent experiments, 6 samples

Supplementary Table S10. Related to Figure 7C. Statistical analysis of IHC fluorescence of PSD-95 puncta in scrambled control and α7-KD hippocampal brain slices in 8 weeks old mice

**Dunnett's multiple comparisons test**	**Mean Diff.**	**95.00% CI of diff.**	**Below threshold?**	**Summary**	**Adjusted P Value**
untreated controls vs. α7 scrambled AAV	1.000	-3.278 to 5.278	No	ns	0.8088
untreated controls vs. α7 shRNA AAV	41.30	37.02 to 45.58	Yes	****	<0.0001

One-way ANOVA followed by Post-hoc-Tukey's multiple comparison test
n refers to the analysis of PSD-95 fluorescence intensity within hippocampal brain slices, ROIs derived from 20 separate images from 5 independent experiments, 6 samples

Supplementary Table S11. Related to Figure 7D. Statistical analysis of IHC fluorescence of SYT puncta in scrambled control and α7-KD hippocampal brain slices in 8 weeks old mice

**Dunnett's multiple comparisons test**	**Mean Diff.**	**95.00% CI of diff.**	**Below threshold?**	**Summary**	**Adjusted P Value**
untreated controls vs. α7 scrambled AAV	-0.1667	-3.574 to 3.241	No	ns	0.9907
untreated controls vs. α7 shRNA AAV	-22.58	-25.99 to -19.18	Yes	****	<0.0001

One-way ANOVA followed by Dunnett's multiple comparison test
n refers to the analysis of SYT fluorescence intensity within hippocampal brain slices, ROIs derived from 20 separate images from 5 independent experiments, 6 samples

Supplementary Table S12. Related to Figure 8A. Mean comparison of degree of colocalization of NFL with GFP in mouse hippocampus

**One sample t test**				
**t, df**	t=7.821, df=9	t=8.162, df=9	t=12.88, df=9	t=9.362, df=9
**P value (two tailed)**	<0.0001	<0.0001	<0.0001	<0.0001
**P value summary**	****	****	****	****
**Significant (alpha=0.05)?**	Yes	Yes	Yes	Yes

One sample t-test
n refers to the analysis of NFL and GFP D.O.C. in mouse brain CA1 region, ROIs derived from 35 separate images from 6 independent experiments

Supplementary Table S13. Related to Figure 8C. Statistical analysis of ICC fluorescence of menin-C in hippocampal neuronal cultures DIV 3-20 in α7 nAChR scrambled AAV, α7 nAChR shRNA AAV, α7 nAChR scrambled AAV+MEN1, α7 nAChR AAV+MEN1AAV and α7 nAChR AAV+GFP AAV neuronal cultures compared to relevant controls

Tukey's multiple comparisons test	Mean Diff.	95.00% CI of diff.	Below threshold?	Summary	Adjusted P Value
Row 1					
α7 scrambled AAV vs. α7 shRNA AAV	-8.417	-14.67 to -2.165	Yes	**	0.0024
α7 scrambled AAV vs. α7 shRNA+GFP	-6.000	-12.25 to 0.2519	No	ns	0.0668
α7 scrambled AAV vs. α7 scrambled+MEN1 AAV	-4.083	-10.34 to 2.169	No	ns	0.3794
α7 scrambled AAV vs. α7 shRNA+MEN1 AAV	-10.42	-16.67 to -4.165	Yes	****	<0.0001
α7 shRNA AAV vs. α7 shRNA+GFP	2.417	-3.835 to 8.669	No	ns	0.8261
α7 shRNA AAV vs. α7 scrambled+MEN1 AAV	4.333	-1.919 to 10.59	No	ns	0.3180
α7 shRNA AAV vs. α7 shRNA+MEN1 AAV	-2.000	-8.252 to 4.252	No	ns	0.9047
α7 shRNA+GFP vs. α7 scrambled+MEN1 AAV	1.917	-4.335 to 8.169	No	ns	0.9173
α7 shRNA+GFP vs. α7 shRNA+MEN1 AAV	-4.417	-10.67 to 1.835	No	ns	0.2988
Row 2					
α7 scrambled AAV vs. α7 shRNA AAV	-25.83	-32.09 to -19.58	Yes	****	<0.0001
α7 scrambled AAV vs. α7 shRNA+GFP	-26.92	-33.17 to -20.66	Yes	****	<0.0001
α7 scrambled AAV vs. α7 scrambled+MEN1 AAV	-29.75	-36.00 to -23.50	Yes	****	<0.0001
α7 scrambled AAV vs. α7 shRNA+MEN1 AAV	-24.75	-31.00 to -18.50	Yes	****	<0.0001
α7 shRNA AAV vs. α7 shRNA+GFP	-1.083	-7.335 to 5.169	No	ns	0.9895
α7 shRNA AAV vs. α7 scrambled+MEN1 AAV	-3.917	-10.17 to 2.335	No	ns	0.4232
α7 shRNA AAV vs. α7 shRNA+MEN1 AAV	1.083	-5.169 to 7.335	No	ns	0.9895

α7 shRNA+GFP vs. α7 scrambled+MEN1 AAV	-2.833	-9.085 to 3.419	No	ns	0.7253
α7 shRNA+GFP vs. α7 shRNA+MEN1 AAV	2.167	-4.085 to 8.419	No	ns	0.8762
α7 scrambled+MEN1 AAV vs. α7 shRNA+MEN1 AAV	5.000	-1.252 to 11.25	No	ns	0.1842
Row 2					
α7 scrambled AAV vs. α7 shRNA AAV	-25.83	-32.09 to -19.58	Yes	****	<0.0001
α7 scrambled AAV vs. α7 shRNA+GFP	-26.92	-33.17 to -20.66	Yes	****	<0.0001
α7 scrambled AAV vs. α7 scrambled+MEN1 AAV	-29.75	-36.00 to -23.50	Yes	****	<0.0001
α7 scrambled AAV vs. α7 shRNA+MEN1 AAV	-24.75	-31.00 to -18.50	Yes	****	<0.0001
α7 shRNA AAV vs. α7 shRNA+GFP	-1.083	-7.335 to 5.169	No	ns	0.9895
Row 3					
α7 scrambled AAV vs. α7 shRNA AAV	-31.25	-37.50 to -25.00	Yes	****	<0.0001
α7 scrambled AAV vs. α7 shRNA+GFP	-32.33	-38.59 to -26.08	Yes	****	<0.0001
α7 scrambled AAV vs. α7 scrambled+MEN1 AAV	-32.58	-38.84 to -26.33	Yes	****	<0.0001
α7 scrambled AAV vs. α7 shRNA+MEN1 AAV	-23.25	-29.50 to -17.00	Yes	****	<0.0001
α7 shRNA AAV vs. α7 shRNA+GFP	-1.083	-7.335 to 5.169	No	ns	0.9895
α7 shRNA AAV vs. α7 scrambled+MEN1 AAV	-1.333	-7.585 to 4.919	No	ns	0.9771
α7 shRNA AAV vs. α7 shRNA+MEN1 AAV	8.000	1.748 to 14.25	Yes	**	0.0047
α7 shRNA+GFP vs. α7 scrambled+MEN1 AAV	-0.2500	-6.502 to 6.002	No	ns	>0.9999

α7 shRNA+GFP vs. α7 shRNA+MEN1 AAV	9.083	2.831 to 15.34	Yes	***	0.0008
α7 scrambled+MEN1 AAV vs. α7 shRNA+MEN1 AAV	9.333	3.081 to 15.59	Yes	***	0.0005
Row 3					
α7 scrambled AAV vs. α7 shRNA AAV	-31.25	-37.50 to -25.00	Yes	****	<0.0001
α7 scrambled AAV vs. α7 shRNA+GFP	-32.33	-38.59 to -26.08	Yes	****	<0.0001
α7 scrambled AAV vs. α7 scrambled+MEN1 AAV	-32.58	-38.84 to -26.33	Yes	****	<0.0001
α7 scrambled AAV vs. α7 shRNA+MEN1 AAV	-23.25	-29.50 to -17.00	Yes	****	<0.0001
α7 shRNA AAV vs. α7 shRNA+GFP	-1.083	-7.335 to 5.169	No	ns	0.9895
Row 4					
α7 scrambled AAV vs. α7 shRNA AAV	-18.58	-24.84 to -12.33	Yes	****	<0.0001
α7 scrambled AAV vs. α7 shRNA+GFP	-15.75	-22.00 to -9.498	Yes	****	<0.0001
α7 scrambled AAV vs. α7 scrambled+MEN1 AAV	-35.83	-42.09 to -29.58	Yes	****	<0.0001
α7 scrambled AAV vs. α7 shRNA+MEN1 AAV	-20.00	-26.25 to -13.75	Yes	****	<0.0001
α7 shRNA AAV vs. α7 shRNA+GFP	2.833	-3.419 to 9.085	No	ns	0.7253
α7 shRNA AAV vs. α7 scrambled+MEN1 AAV	-17.25	-23.50 to -11.00	Yes	****	<0.0001
α7 shRNA AAV vs. α7 shRNA+MEN1 AAV	-1.417	-7.669 to 4.835	No	ns	0.9714
α7 shRNA+GFP vs. α7 scrambled+MEN1 AAV	-20.08	-26.34 to -13.83	Yes	****	<0.0001
α7 shRNA+GFP vs. α7 shRNA+MEN1 AAV	-4.250	-10.50 to 2.002	No	ns	0.3379

α7 scrambled+MEN1 AAV vs. α7 shRNA+MEN1 AAV	15.83	9.581 to 22.09	Yes	****	<0.0001
Row 4					
α7 scrambled AAV vs. α7 shRNA AAV	-18.58	-24.84 to -12.33	Yes	****	<0.0001
α7 scrambled AAV vs. α7 shRNA+GFP	-15.75	-22.00 to -9.498	Yes	****	<0.0001
α7 scrambled AAV vs. α7 scrambled+MEN1 AAV	-35.83	-42.09 to -29.58	Yes	****	<0.0001
α7 scrambled AAV vs. α7 shRNA+MEN1 AAV	-20.00	-26.25 to -13.75	Yes	****	<0.0001
α7 shRNA AAV vs. α7 shRNA+GFP	2.833	-3.419 to 9.085	No	ns	0.7253
α7 shRNA AAV vs. α7 scrambled+MEN1 AAV	-17.25	-23.50 to -11.00	Yes	****	<0.0001
α7 shRNA AAV vs. α7 shRNA+MEN1 AAV	-1.417	-7.669 to 4.835	No	ns	0.9714
α7 shRNA+GFP vs. α7 scrambled+MEN1 AAV	-20.08	-26.34 to -13.83	Yes	****	<0.0001
α7 shRNA+GFP vs. α7 shRNA+MEN1 AAV	-4.250	-10.50 to 2.002	No	ns	0.3379
Row 5					
α7 scrambled AAV vs. α7 shRNA AAV	39.00	32.75 to 45.25	Yes	****	<0.0001
α7 scrambled AAV vs. α7 shRNA+GFP	41.67	35.41 to 47.92	Yes	****	<0.0001
α7 scrambled AAV vs. α7 scrambled+MEN1 AAV	-51.83	-58.09 to -45.58	Yes	****	<0.0001
α7 scrambled AAV vs. α7 shRNA+MEN1 AAV	-20.25	-26.50 to -14.00	Yes	****	<0.0001
α7 shRNA AAV vs. α7 shRNA+GFP	2.667	-3.585 to 8.919	No	ns	0.7678
α7 shRNA AAV vs. α7 scrambled+MEN1 AAV	-90.83	-97.09 to -84.58	Yes	****	<0.0001

α7 shRNA AAV vs. α7 shRNA+MEN1 AAV	-59.25	-65.50 to - 53.00	Yes	****	<0.0001
α7 shRNA+GFP vs. α7 scrambled+MEN1 AAV	-93.50	-99.75 to - 87.25	Yes	****	<0.0001
α7 shRNA+GFP vs. α7 shRNA+MEN1 AAV	-61.92	-68.17 to - 55.66	Yes	****	<0.0001
α7 scrambled+MEN1 AAV vs. α7 shRNA+MEN1 AAV	31.58	25.33 to 37.84	Yes	****	<0.0001
Row 5					
α7 scrambled AAV vs. α7 shRNA AAV	39.00	32.75 to 45.25	Yes	****	<0.0001
α7 scrambled AAV vs. α7 shRNA+GFP	41.67	35.41 to 47.92	Yes	****	<0.0001
α7 scrambled AAV vs. α7 scrambled+MEN1 AAV	-51.83	-58.09 to - 45.58	Yes	****	<0.0001
α7 scrambled AAV vs. α7 shRNA+MEN1 AAV	-20.25	-26.50 to - 14.00	Yes	****	<0.0001
α7 shRNA AAV vs. α7 shRNA+GFP	2.667	-3.585 to 8.919	No	ns	0.7678
α7 shRNA AAV vs. α7 scrambled+MEN1 AAV	-90.83	-97.09 to - 84.58	Yes	****	<0.0001
α7 shRNA AAV vs. α7 shRNA+MEN1 AAV	-59.25	-65.50 to - 53.00	Yes	****	<0.0001
α7 shRNA+GFP vs. α7 scrambled+MEN1 AAV	-93.50	-99.75 to - 87.25	Yes	****	<0.0001
α7 shRNA+GFP vs. α7 shRNA+MEN1 AAV	-61.92	-68.17 to - 55.66	Yes	****	<0.0001
α7 scrambled+MEN1 AAV vs. α7 shRNA+MEN1 AAV	31.58	25.33 to 37.84	Yes	****	<0.0001

Two-way ANOVA followed by Post-hoc-Tukey’s multiple comparison test
n refers to the analysis of menin-C puncta fluorescence intensity within neuronal cultures, ROIs derived from 25 separate images from 5 independent experiments, 7 samples

Supplementary Table S14. Related to Figure 8D. Statistical analysis of IHC fluorescence of menin-C in $\alpha7$ nAChR scrambled AAV, $\alpha7$ nAChR shRNA AAV, $\alpha7$ nAChR scrambled AAV+MEN1, $\alpha7$ nAChR AAV+MEN1AAV and $\alpha7$ nAChR AAV+GFP AAV CA1 hippocampal slices compared to relevant controls

**Šídák's multiple comparisons test**	**Mean Diff.**	**95.00% CI of diff.**	**Below threshold ?**	**Summary**	**Adjusted P Value**
α7 scrambled AAV vs. α7 shRNA AAV	55.80	48.01 to 63.59	Yes	****	<0.0001
α7 scrambled AAV vs. α7 scrambled +MEN1 AAV	-32.90	-40.69 to -25.11	Yes	****	<0.0001
α7 scrambled AAV vs. α7 shRNA+MEN1 AAV	1.800	-5.986 to 9.586	No	ns	0.9990
α7 scrambled AAV vs. α7 shRNA+GFP	52.90	45.11 to 60.69	Yes	****	<0.0001
α7 shRNA AAV vs. α7 scrambled +MEN1 AAV	-88.70	-96.49 to -80.91	Yes	****	<0.0001
α7 shRNA AAV vs. α7 shRNA+MEN1 AAV	-54.00	-61.79 to -46.21	Yes	****	<0.0001
α7 shRNA AAV vs. α7 shRNA+GFP	-2.900	-10.69 to 4.886	No	ns	0.9619
α7 scrambled +MEN1 AAV vs. α7 shRNA+MEN1 AAV	34.70	26.91 to 42.49	Yes	****	<0.0001
α7 scrambled +MEN1 AAV vs. α7 shRNA+GFP	85.80	78.01 to 93.59	Yes	****	<0.0001
α7 shRNA+MEN1 AAV vs. α7 shRNA+GFP	51.10	43.31 to 58.89	Yes	****	<0.0001
Šídák's multiple comparisons test	Mean Diff.	95.00% CI of diff.	Below threshold?	Summary	Adjusted P Value
α7 scrambled AAV vs. α7 shRNA AAV	55.80	48.01 to 63.59	Yes	****	<0.0001
α7 scrambled AAV vs. α7 scrambled +MEN1 AAV	-32.90	-40.69 to -25.11	Yes	****	<0.0001
α7 scrambled AAV vs. α7 shRNA+MEN1 AAV	1.800	-5.986 to 9.586	No	ns	0.9990
α7 scrambled AAV vs. α7 shRNA+GFP	52.90	45.11 to 60.69	Yes	****	<0.0001
α7 shRNA AAV vs. α7 scrambled +MEN1 AAV	-88.70	-96.49 to -80.91	Yes	****	<0.0001
α7 shRNA AAV vs. α7 shRNA+MEN1 AAV	-54.00	-61.79 to -46.21	Yes	****	<0.0001

α7 shRNA AAV vs. α7 shRNA+GFP	- 2.900	-10.69 to 4.886	No	ns	0.9619
α7 scrambled +MEN1 AAV vs. α7 shRNA+MEN1 AAV	34.70	26.91 to 42.49	Yes	****	<0.0001
α7 scrambled +MEN1 AAV vs. α7 shRNA+GFP	85.80	78.01 to 93.59	Yes	****	<0.0001
α7 shRNA+MEN1 AAV vs. α7 shRNA+GFP	51.10	43.31 to 58.89	Yes	****	<0.0001

Two-way ANOVA followed by Sidak's multiple comparison test
n refers to the analysis of menin-C puncta fluorescence intensity within neuronal cultures, ROIs derived from 30 separate images from 5 independent experiments, 7 samples

Supplementary Table S15. Related to Figure 9A. Statistical analysis of qPCR analysis of Multiple endocrine neoplasia type 1, *MEN1* gene, nAChRs neuronal subunits *α*2-7 and β2-4 in *α*7 nAChR scrambled AAV, *α*7 nAChR shRNA AAV, *α*7 nAChR scrambled AAV+MEN1, *α*7 nAChR AAV+MEN1AAV and *α*7 nAChR AAV+GFP AAV hippocampal neuronal cultures

**Šídák's multiple comparisons test**	**Mean Diff.**	**95.00% CI of diff.**	**Below threshold?**	**Summary**	**Adjusted P Value**
sca7men vs. sca7a7	-0.02612	-0.3943 to 0.3420	No	ns	>0.9999
sca7men vs. sha7men	1.091	0.8959 to 1.287	Yes	***	0.0003
sca7men vs. sha7a7	1.103	0.8114 to 1.394	Yes	***	0.0010
sca7men vs. sca7+MEn1men	-1.252	-1.485 to -1.020	Yes	***	0.0003
sca7men vs. sca7+MEn1a7	-1.907	-2.479 to -1.335	Yes	**	0.0014
sca7men vs. shRNA7+GFPmen	1.100	0.8731 to 1.328	Yes	***	0.0005
sca7men vs. shRNA7+GFPa7	1.105	0.8763 to 1.333	Yes	***	0.0005
sca7men vs. shRNA7+MEn1men	0.01587	-0.1935 to 0.2252	No	ns	>0.9999
sca7men vs. shRNA7+MEn1a7	-0.1539	-0.6023 to 0.2946	No	ns	0.6644
sca7a7 vs. sha7men	1.118	0.7228 to 1.512	Yes	**	0.0023

sca7a7 vs. sha7a7	1.129	0.6877 to 1.570	Yes	**	0.0031
sca7a7 vs. sca7+MEn1men	-1.226	-1.788 to -0.6640	Yes	**	0.0050
sca7a7 vs. sca7+MEn1a7	-1.881	-2.684 to -1.078	Yes	**	0.0041
sca7a7 vs. shRNA7+GFPmen	1.127	0.7172 to 1.536	Yes	**	0.0025
sca7a7 vs. shRNA7+GFPa7	1.131	0.7185 to 1.543	Yes	**	0.0025
sca7a7 vs. shRNA7+MEn1men	0.04199	-0.3540 to 0.4379	No	ns	>0.9999
sca7a7 vs. shRNA7+MEn1a7	-0.1277	-0.3408 to 0.08534	No	ns	0.2047
sha7men vs. sha7a7	0.01119	-0.08651 to 0.1089	No	ns	>0.9999
sha7men vs. sca7+MEn1men	-2.344	-2.543 to -2.144	Yes	****	<0.0001
sha7men vs. sca7+MEn1a7	-2.999	-3.734 to -2.264	Yes	***	0.0008
sha7men vs. shRNA7+GFPmen	0.009032	-0.02286 to 0.04092	No	ns	0.8362
sha7men vs. shRNA7+GFPa7	0.01314	-0.02001 to 0.04629	No	ns	0.5219
sha7men vs. shRNA7+MEn1men	-1.076	-1.095 to -1.056	Yes	****	<0.0001
sha7men vs. shRNA7+MEn1a7	-1.245	-1.779 to -0.7118	Yes	**	0.0041
sha7a7 vs. sca7+MEn1men	-2.355	-2.602 to -2.108	Yes	****	<0.0001
sha7a7 vs. sca7+MEn1a7	-3.010	-3.838 to -2.182	Yes	**	0.0011
sha7a7 vs. shRNA7+GFPmen	-0.002156	-0.06957 to 0.06525	No	ns	>0.9999
sha7a7 vs. shRNA7+GFPa7	0.001949	-0.06351 to 0.06741	No	ns	>0.9999

sha7a7 vs. shRNA7+MEn1men	-1.087	-1.177 to -0.9969	Yes	****	<0.0001
sha7a7 vs. shRNA7+MEn1a7	-1.256	-1.846 to -0.6666	Yes	**	0.0054
sca7+MEn1men vs. sca7+MEn1a7	-0.6551	-1.305 to -0.005092	Yes	*	0.0489
sca7+MEn1men vs. shRNA7+GFPmen	2.353	2.143 to 2.562	Yes	****	<0.0001
sca7+MEn1men vs. shRNA7+GFPa7	2.357	2.149 to 2.565	Yes	****	<0.0001
sca7+MEn1men vs. shRNA7+MEn1men	1.268	1.060 to 1.476	Yes	***	0.0002
sca7+MEn1men vs. shRNA7+MEn1a7	1.098	0.4308 to 1.766	Yes	*	0.0116
sca7+MEn1a7 vs. shRNA7+GFPmen	3.008	2.245 to 3.770	Yes	***	0.0009
sca7+MEn1a7 vs. shRNA7+GFPa7	3.012	2.248 to 3.776	Yes	***	0.0009
sca7+MEn1a7 vs. shRNA7+MEn1men	1.923	1.181 to 2.666	Yes	**	0.0030
sca7+MEn1a7 vs. shRNA7+MEn1a7	1.753	0.9397 to 2.567	Yes	**	0.0052
shRNA7+GFPmen vs. shRNA7+GFPa7	0.004104	-0.0004204 to 0.008629	No	ns	0.0661
shRNA7+GFPmen vs. shRNA7+MEn1men	-1.085	-1.108 to -1.061	Yes	****	<0.0001
shRNA7+GFPmen vs. shRNA7+MEn1a7	-1.254	-1.808 to -0.7002	Yes	**	0.0045
shRNA7+GFPa7 vs. shRNA7+MEn1men	-1.089	-1.116 to -1.061	Yes	****	<0.0001
shRNA7+GFPa7 vs. shRNA7+MEn1a7	-1.258	-1.814 to -0.7027	Yes	**	0.0045
shRNA7+MEn1men vs. shRNA7+MEn1a7	-0.1697	-0.7111 to 0.3716	No	ns	0.7505
Šídák's multiple comparisons test	Mean Diff.	95.00% CI of diff.	Below threshold?	Summary	Adjusted P Value

sca7men vs. sca7a7	-0.02612	-0.3943 to 0.3420	No	ns	>0.9999
sca7men vs. sha7men	1.091	0.8959 to 1.287	Yes	***	0.0003
sca7men vs. sha7a7	1.103	0.8114 to 1.394	Yes	***	0.0010
sca7men vs. sca7+MEn1men	-1.252	-1.485 to -1.020	Yes	***	0.0003
sca7men vs. sca7+MEn1a7	-1.907	-2.479 to -1.335	Yes	**	0.0014
sca7men vs. shRNA7+GFPmen	1.100	0.8731 to 1.328	Yes	***	0.0005
sca7men vs. shRNA7+GFPa7	1.105	0.8763 to 1.333	Yes	***	0.0005
sca7men vs. shRNA7+MEn1men	0.01587	-0.1935 to 0.2252	No	ns	>0.9999
sca7men vs. shRNA7+MEn1a7	-0.1539	-0.6023 to 0.2946	No	ns	0.6644
sca7a7 vs. sha7men	1.118	0.7228 to 1.512	Yes	**	0.0023
sca7a7 vs. sha7a7	1.129	0.6877 to 1.570	Yes	**	0.0031
sca7a7 vs. sca7+MEn1men	-1.226	-1.788 to -0.6640	Yes	**	0.0050
sca7a7 vs. sca7+MEn1a7	-1.881	-2.684 to -1.078	Yes	**	0.0041
sca7a7 vs. shRNA7+GFPmen	1.127	0.7172 to 1.536	Yes	**	0.0025
sca7a7 vs. shRNA7+GFPa7	1.131	0.7185 to 1.543	Yes	**	0.0025
sca7a7 vs. shRNA7+MEn1men	0.04199	-0.3540 to 0.4379	No	ns	>0.9999
sca7a7 vs. shRNA7+MEn1a7	-0.1277	-0.3408 to 0.08534	No	ns	0.2047
sha7men vs. sha7a7	0.01119	-0.08651 to 0.1089	No	ns	>0.9999

sha7men vs. sca7+MEn1men	-2.344	-2.543 to -2.144	Yes	****	<0.0001
sha7men vs. sca7+MEn1a7	-2.999	-3.734 to -2.264	Yes	***	0.0008
sha7men vs. shRNA7+GFPmen	0.009032	-0.02286 to 0.04092	No	ns	0.8362
sha7men vs. shRNA7+GFPa7	0.01314	-0.02001 to 0.04629	No	ns	0.5219
sha7men vs. shRNA7+MEn1men	-1.076	-1.095 to -1.056	Yes	****	<0.0001
sha7men vs. shRNA7+MEn1a7	-1.245	-1.779 to -0.7118	Yes	**	0.0041
sha7a7 vs. sca7+MEn1men	-2.355	-2.602 to -2.108	Yes	****	<0.0001
sha7a7 vs. sca7+MEn1a7	-3.010	-3.838 to -2.182	Yes	**	0.0011
sha7a7 vs. shRNA7+GFPmen	-0.002156	-0.06957 to 0.06525	No	ns	>0.9999
sha7a7 vs. shRNA7+GFPa7	0.001949	-0.06351 to 0.06741	No	ns	>0.9999
sha7a7 vs. shRNA7+MEn1men	-1.087	-1.177 to -0.9969	Yes	****	<0.0001
sha7a7 vs. shRNA7+MEn1a7	-1.256	-1.846 to -0.6666	Yes	**	0.0054
sca7+MEn1men vs. sca7+MEn1a7	-0.6551	-1.305 to -0.005092	Yes	*	0.0489
sca7+MEn1a7 vs. shRNA7+GFPmen	3.008	2.245 to 3.770	Yes	***	0.0009
sca7+MEn1a7 vs. shRNA7+GFPa7	3.012	2.248 to 3.776	Yes	***	0.0009
sca7+MEn1a7 vs. shRNA7+MEn1men	1.923	1.181 to 2.666	Yes	**	0.0030
sca7+MEn1a7 vs. shRNA7+MEn1a7	1.753	0.9397 to 2.567	Yes	**	0.0052
shRNA7+GFPmen vs. shRNA7+GFPa7	0.004104	-0.0004204 to 0.008629	No	ns	0.0661

shRNA7+GFPmen vs. shRNA7+MEn1men	-1.085	-1.108 to -1.061	Yes	****	<0.0001
shRNA7+GFPmen vs. shRNA7+MEn1a7	-1.254	-1.808 to -0.7002	Yes	**	0.0045
shRNA7+GFPa7 vs. shRNA7+MEn1men	-1.089	-1.116 to -1.061	Yes	****	<0.0001
shRNA7+GFPa7 vs. shRNA7+MEn1a7	-1.258	-1.814 to -0.7027	Yes	**	0.0045
shRNA7+MEn1men vs. shRNA7+MEn1a7	-0.1697	-0.7111 to 0.3716	No	ns	0.7505

One-way ANOVA followed by Post-hoc-Tukey's multiple comparison test
qPCR (n=6, 3 independent experiments each, triplicate replicates)

Supplementary Table S16. Related to Figure 9C. Statistical analysis of ICC fluorescence of PSD-95 in hippocampal neuronal cultures DIV 3-20 in *α*7 nAChR scrambled AAV, *α*7 nAChR shRNA AAV, *α*7 nAChR scrambled AAV+MEN1, *α*7 nAChR AAV+MEN1AAV and *α*7 nAChR AAV+GFP AAV treated hippocampal neuronal cultures

**Tukey's multiple comparisons test**	**Mean Diff.**	**95.00% CI of diff.**	**Below threshold?**	**Summary**	**Adjusted P Value**
Row 1					
α7 scrambled AAV vs. α7 shRNA AAV	4.833	0.3414 to 9.325	Yes	*	0.0279
α7 scrambled AAV vs. α7 scrambled+MEN1 AAV	-2.167	-6.659 to 2.325	No	ns	0.6762
α7 scrambled AAV vs. α7 shRNA AAV +GFP	7.667	3.175 to 12.16	Yes	****	<0.0001
α7 scrambled AAV vs. α7 shRNA+MEN1 AAV	5.083	0.5914 to 9.575	Yes	*	0.0176
α7 shRNA AAV vs. α7 scrambled+MEN1 AAV	-7.000	-11.49 to -2.508	Yes	***	0.0002

α7 shRNA AAV vs. α7 shRNA AAV +GFP	2.833	-1.659 to 7.325	No	ns	0.4160
α7 shRNA AAV vs. α7 shRNA+MEN1 AAV	0.2500	-4.242 to 4.742	No	ns	0.9999
Row 2					
α7 scrambled AAV vs. α7 shRNA AAV	56.92	52.42 to 61.41	Yes	****	<0.0001
α7 scrambled AAV vs. α7 scrambled+MEN1 AAV	-12.08	-16.58 to -7.591	Yes	****	<0.0001
α7 scrambled AAV vs. α7 shRNA AAV +GFP	54.50	50.01 to 58.99	Yes	****	<0.0001
α7 scrambled AAV vs. α7 shRNA+MEN1 AAV	15.00	10.51 to 19.49	Yes	****	<0.0001
α7 shRNA AAV vs. α7 scrambled+MEN1 AAV	-69.00	-73.49 to -64.51	Yes	****	<0.0001
α7 shRNA AAV vs. α7 shRNA AAV +GFP	-2.417	-6.909 to 2.075	No	ns	0.5781
α7 shRNA AAV vs. α7 shRNA+MEN1 AAV	-41.92	-46.41 to -37.42	Yes	****	<0.0001
α7 scrambled+MEN1 AAV vs. α7 shRNA AAV +GFP	66.58	62.09 to 71.08	Yes	****	<0.0001
α7 scrambled+MEN1 AAV vs. α7 shRNA+MEN1 AAV	27.08	22.59 to 31.58	Yes	****	<0.0001
α7 shRNA AAV +GFP vs. α7	-39.50	-43.99 to -35.01	Yes	****	<0.0001

shRNA+MEN1 AAV					
Row 3					
α7 scrambled AAV vs. α7 shRNA AAV	64.25	59.76 to 68.74	Yes	****	<0.0001
α7 scrambled AAV vs. α7 scrambled+MEN1 AAV	-13.50	-17.99 to -9.008	Yes	****	<0.0001
α7 scrambled AAV vs. α7 shRNA AAV +GFP	64.08	59.59 to 68.58	Yes	****	<0.0001
α7 scrambled AAV vs. α7 shRNA+MEN1 AAV	6.417	1.925 to 10.91	Yes	**	0.0010
α7 shRNA AAV vs. α7 scrambled+MEN1 AAV	-77.75	-82.24 to -73.26	Yes	****	<0.0001
α7 shRNA AAV vs. α7 shRNA AAV +GFP	-0.1667	-4.659 to 4.325	No	ns	>0.9999
α7 shRNA AAV vs. α7 shRNA+MEN1 AAV	-57.83	-62.33 to -53.34	Yes	****	<0.0001
α7 scrambled+MEN1 AAV vs. α7 shRNA AAV +GFP	77.58	73.09 to 82.08	Yes	****	<0.0001
α7 scrambled+MEN1 AAV vs. α7 shRNA+MEN1 AAV	19.92	15.42 to 24.41	Yes	****	<0.0001
α7 shRNA AAV +GFP vs. α7 shRNA+MEN1 AAV	-57.67	-62.16 to -53.17	Yes	****	<0.0001
Row 4					

α7 scrambled AAV vs. α7 shRNA AAV	54.42	49.92 to 58.91	Yes	****	<0.0001
α7 scrambled AAV vs. α7 scrambled+MEN1 AAV	-22.17	-26.66 to -17.67	Yes	****	<0.0001
α7 scrambled AAV vs. α7 shRNA AAV +GFP	55.92	51.42 to 60.41	Yes	****	<0.0001
α7 scrambled AAV vs. α7 shRNA+MEN1 AAV	-1.250	-5.742 to 3.242	No	ns	0.9406
α7 shRNA AAV vs. α7 scrambled+MEN1 AAV	-76.58	-81.08 to -72.09	Yes	****	<0.0001
α7 shRNA AAV vs. α7 shRNA AAV +GFP	1.500	-2.992 to 5.992	No	ns	0.8902
α7 shRNA AAV vs. α7 shRNA+MEN1 AAV	-55.67	-60.16 to -51.17	Yes	****	<0.0001
α7 scrambled+MEN1 AAV vs. α7 shRNA AAV +GFP	78.08	73.59 to 82.58	Yes	****	<0.0001
α7 scrambled+MEN1 AAV vs. α7 shRNA+MEN1 AAV	20.92	16.42 to 25.41	Yes	****	<0.0001
α7 shRNA AAV +GFP vs. α7 shRNA+MEN1 AAV	-57.17	-61.66 to -52.67	Yes	****	<0.0001
Row 5					
α7 scrambled AAV vs. α7 shRNA AAV	54.42	49.92 to 58.91	Yes	****	<0.0001
α7 scrambled AAV vs. α7	-19.42	-23.91 to -14.92	Yes	****	<0.0001

scrambled+MEN1 AAV					
α7 scrambled AAV vs. α7 shRNA AAV +GFP	56.42	51.92 to 60.91	Yes	****	<0.0001
α7 scrambled AAV vs. α7 shRNA+MEN1 AAV	-1.083	-5.575 to 3.409	No	ns	0.9642
α7 shRNA AAV vs. α7 scrambled+MEN1 AAV	-73.83	-78.33 to -69.34	Yes	****	<0.0001
α7 shRNA AAV vs. α7 shRNA AAV +GFP	2.000	-2.492 to 6.492	No	ns	0.7382
α7 shRNA AAV vs. α7 shRNA+MEN1 AAV	-55.50	-59.99 to -51.01	Yes	****	<0.0001
α7 scrambled+MEN1 AAV vs. α7 shRNA AAV +GFP	75.83	71.34 to 80.33	Yes	****	<0.0001
α7 scrambled+MEN1 AAV vs. α7 shRNA+MEN1 AAV	18.33	13.84 to 22.83	Yes	****	<0.0001
α7 shRNA AAV +GFP vs. α7 shRNA+MEN1 AAV	-57.50	-61.99 to -53.01	Yes	****	<0.0001

Two-way ANOVA followed by Post-hoc Tukey's multiple comparison test
n refers to the analysis of menin-C puncta fluorescence intensity within neuronal cultures, ROIs derived from 35 separate images from 5 independent experiments, 7 samples

Supplementary Table S17. Related to Figure 10C. Statistical analysis ratio of freezing episodes from day 2 to day 1 in $\alpha7$ nAChR scrambled AAV, $\alpha7$ nAChR shRNA AAV, $\alpha7$ nAChR AAV+MEN1AAV injected mice

Dunnett's multiple comparisons test	Mean Diff.	95.00% CI of diff.	Below threshold?	Summary	Adjusted P Value
α7 scrambled AAV vs. α7 shRNA AAV	47.60	44.42 to 50.78	Yes	****	<0.0001
α7 scrambled AAV vs. α7 shRNA AAV +MEN1	5.933	2.754 to 9.113	Yes	***	0.0002

Ono-way ANOVA followed by Dunette's multiple comparison test
n refers to 15 animals per group

Supplementary Table S18. Related to Figure S6. Statistical analysis ratio of freezing percentages 1 in $\alpha7$ nAChR scrambled AAV, $\alpha7$ nAChR shRNA AAV, $\alpha7$ nAChR scrambled AAV,+MEN1 AAV, $\alpha7$ nAChR AAV+MEN1AAV injected mice

Dunnett's multiple comparisons test	Mean Diff.	95.00% CI of diff.	Below threshold?	Summary	Adjusted P Value
α7 scrambled AAV vs. α7 shRNA AAV	46.33	42.38 to 50.29	Yes	****	<0.0001
α7 scrambled AAV vs. α7 shRNA AAV +MEN1	2.867	-1.091 to 6.824	No	ns	0.2043
α7 scrambled AAV vs. α7 scrambled +MEN1 AAV	-0.9190	-4.946 to 3.108	No	ns	0.9034

Ono-way ANOVA followed by Dunette's multiple comparison test
n refers to 15 animals per group

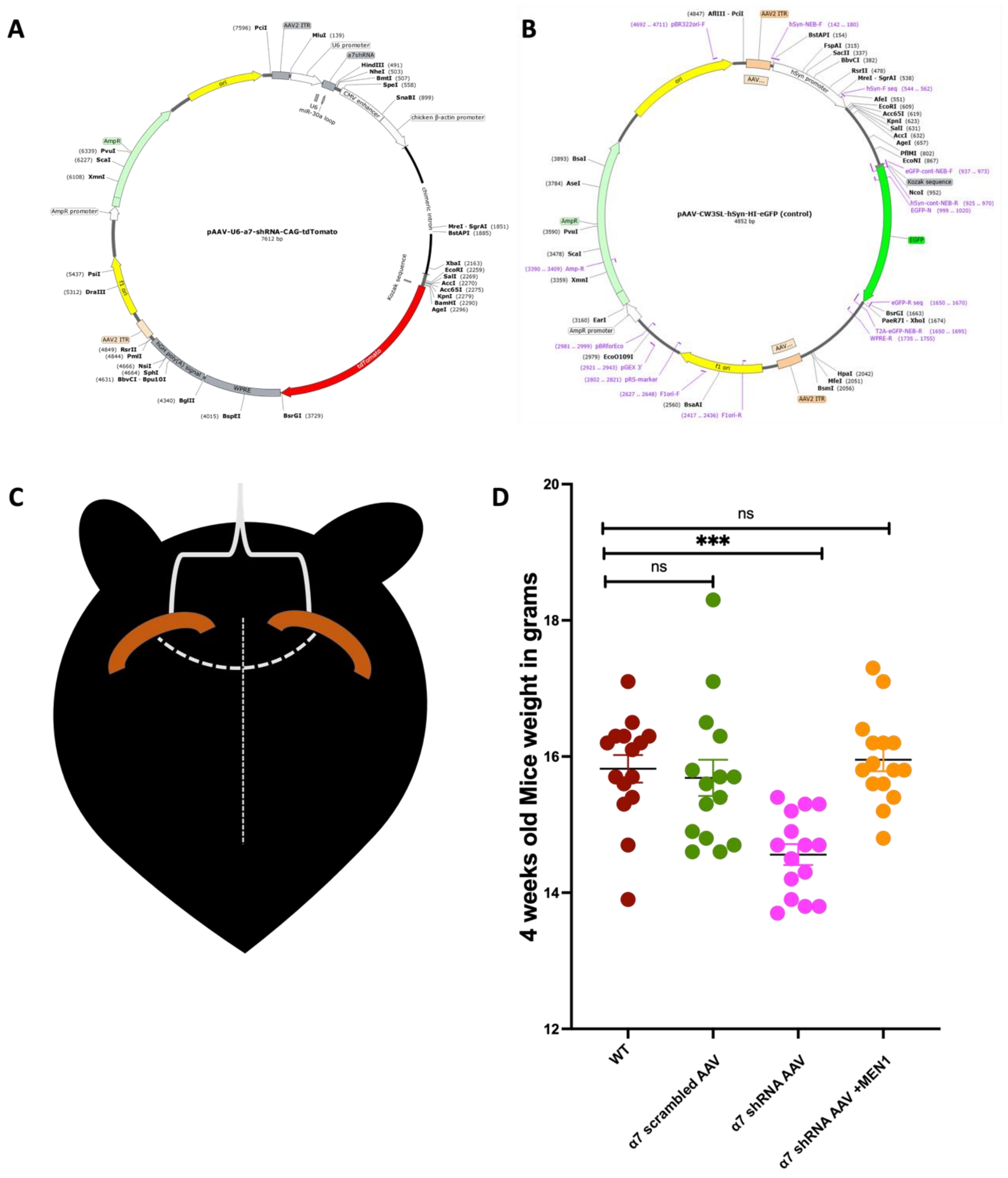

FIGURE S1

AAV plasmid maps for α7 KD and MEN1 encoding AAV. (A) plamid map for α7 shRNA AAV and its scrambled control (B) plasmid map for MEN1 encoding AAV and GFP only control (C) illustrative diagram for stereotaxic viral administration into CA1 specific coordinates. (D) Summary data, mice weights in grams in Wild type, α7 shRNA AAV and its scrambled control animals, α7 shRNA AAV+ MEN1 AAV. Statistical significance (One-way ANOVA followed by Tukey's multiple comparison test) ***$p < 0.001$, ns $P > 0.05$.

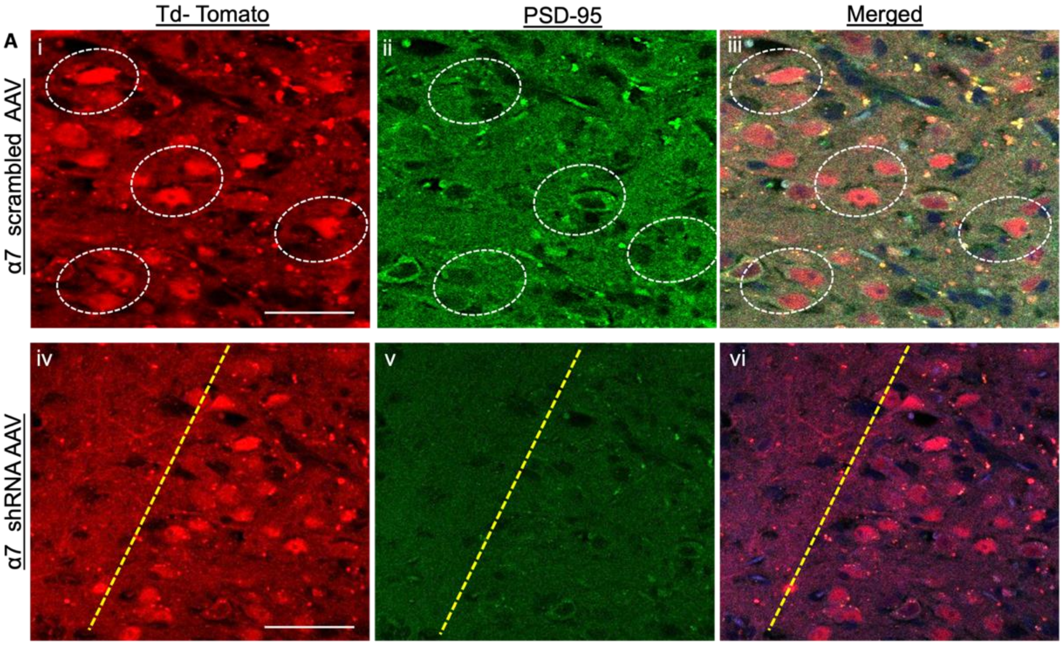

FIGURE S2

PSD-95 expression reduces in α7 KD hippocampal slices *(in-vivo)*. (A) IHC characterization of α7 KD hippocampal slices (Aiv-vi) 8 weeks old. mice (n = 20 images, 5 independent samples, representative image) compared to scrambled controls (Ai-iii). α7 KD hippocampal slices labelled td-tomato positive neurons (Ai,iv) labelled with PSD-95 (postsynaptic marker) (Aii,v) and merged (Aiii,vi) exhibiting reduced expression of PSD-95 in CA1 neurons.

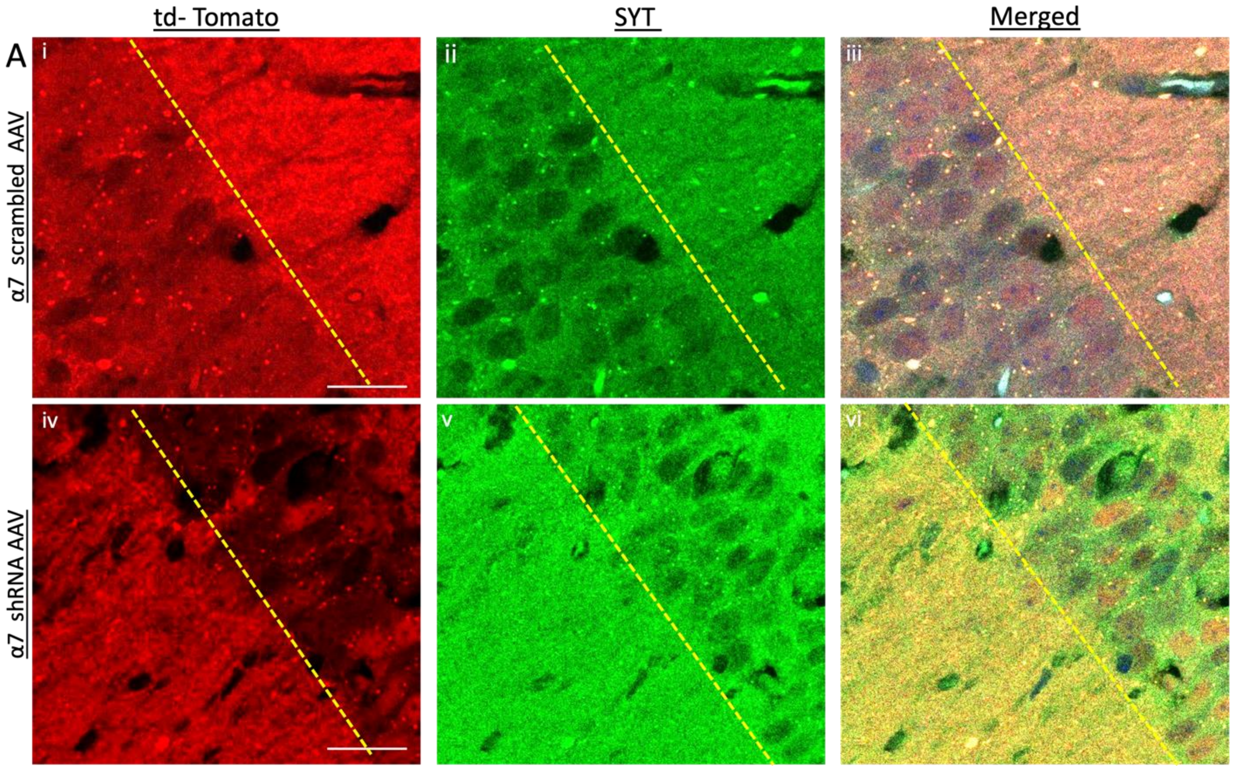

FIGURE S3

SYT expression increases in α7 KD hippocampal slices *(in-vivo)*. (A) IHC characterization of α7 KD hippocampal slices (Aiv-vi) 8 weeks old. mice (n = 20 images, 5 independent samples, representative image) compared to scrambled controls (Ai-iii). α7 KD hippocampal slices labelled td-tomato positive neurons (Ai,iv) labelled with SYT (presynaptic marker) (Aii,v) and merged (Aiii,vi) exhibiting increased expression of PSD-95 in CA1 neurons.

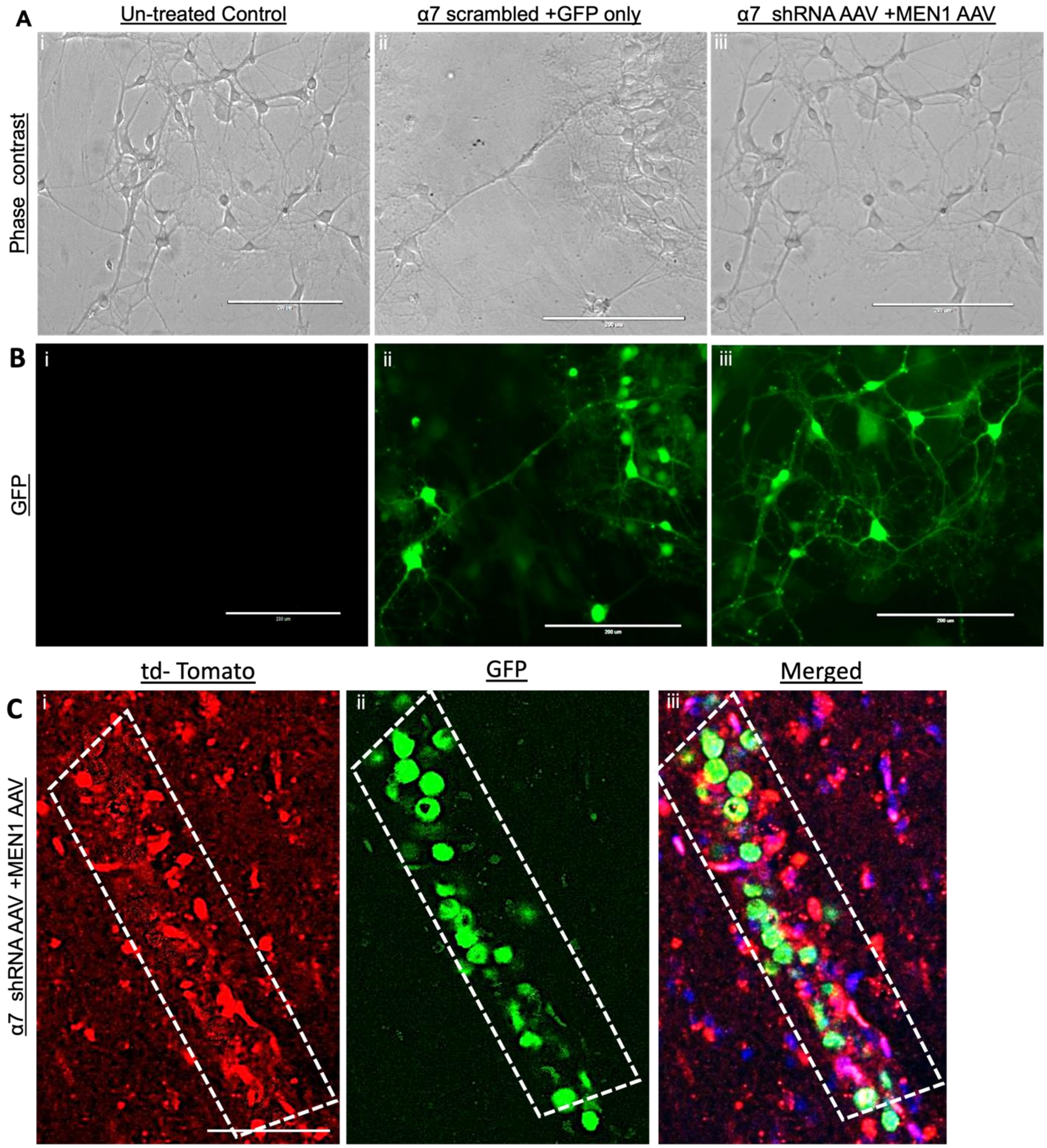

FIGURE S4

Co-expression of α7 shRNA AAV and MEN1 encoding AAV. (A) live cell phase contrast (i-iii), (B) GFP fluorescence (i-iii); images of untreated control (Ai,Bi), scrambled control (Aii, Bii) and MEN1 encoding AAV (Aiii,Biii) AAV transduced hippocampal cultures at DIV 7 (n=21 images, 7 independent samples each, representative images). (C) Co expression of tdTomato and GFP in the CA1 specific neurons. Scale bar (A-B)200μm, (C)75 μm

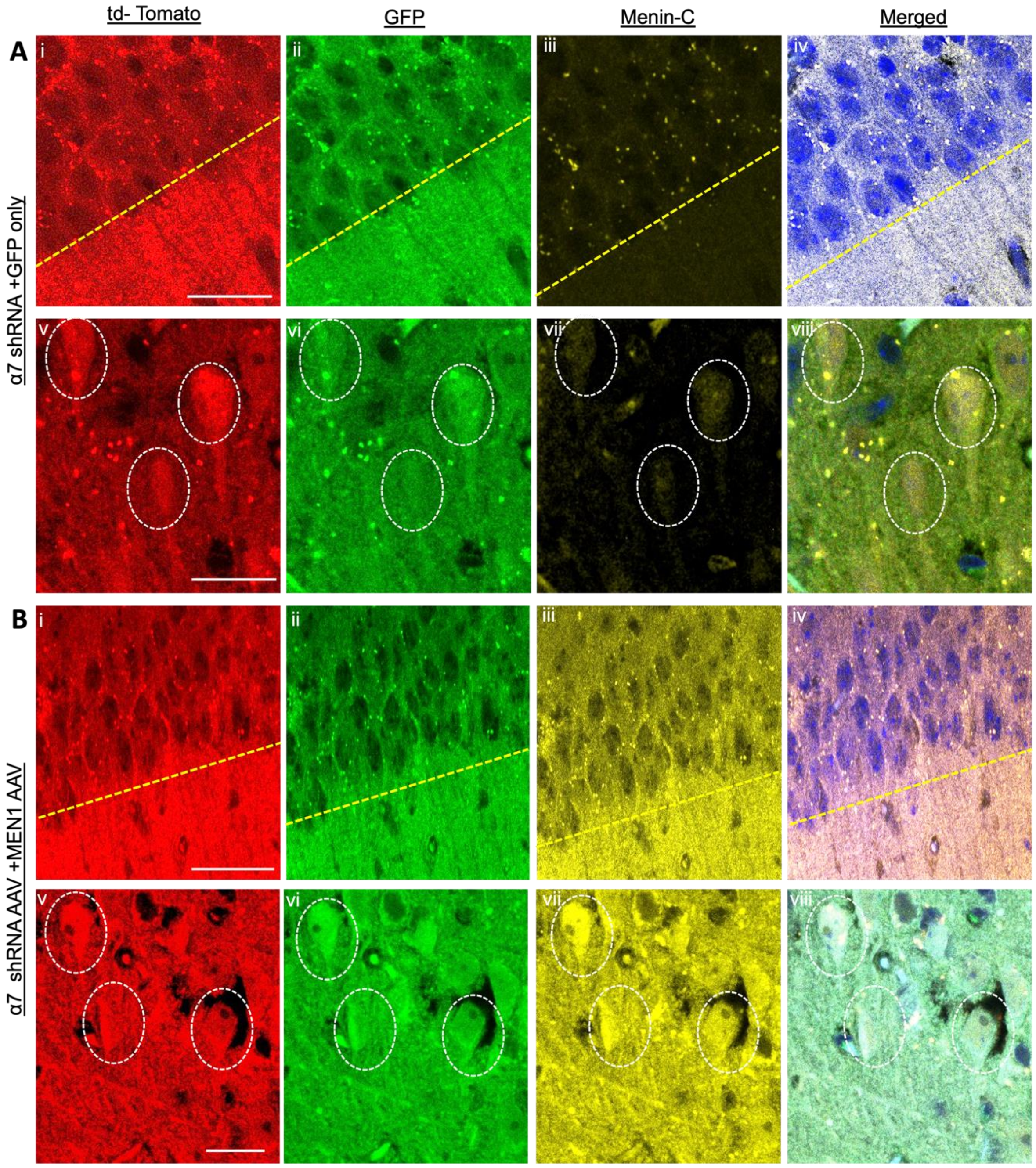

FIGURE S5

Menin-C expression reduces in α7 KD hippocampal slices *(in-vivo)*. (A) IHC characterization of α7 KD hippocampal slices (Ai-viii) 12 weeks old. mice (n = 20 images, 5 independent samples, representative image). α7 KD+GFP only hippocampal slices labelled td-tomato positive (Ai,v)+GFP positive neurons (Aii,vi) labelled with menin-C (Aiii,vii) and merged (Aiv,viii), (Av-viii) zoomed in CA1 neurons exhibiting reduced menin-C expression. α7 KD+MEN1 encoding AAV hippocampal slices labelled td-tomato positive (Ai,v)+GFP positive neurons (Aii,vi) labelled with menin-C (Aiii,vii) and merged (Aiv,viii), (Av-viii) zoomed in CA1 neurons exhibiting increased menin-C expression

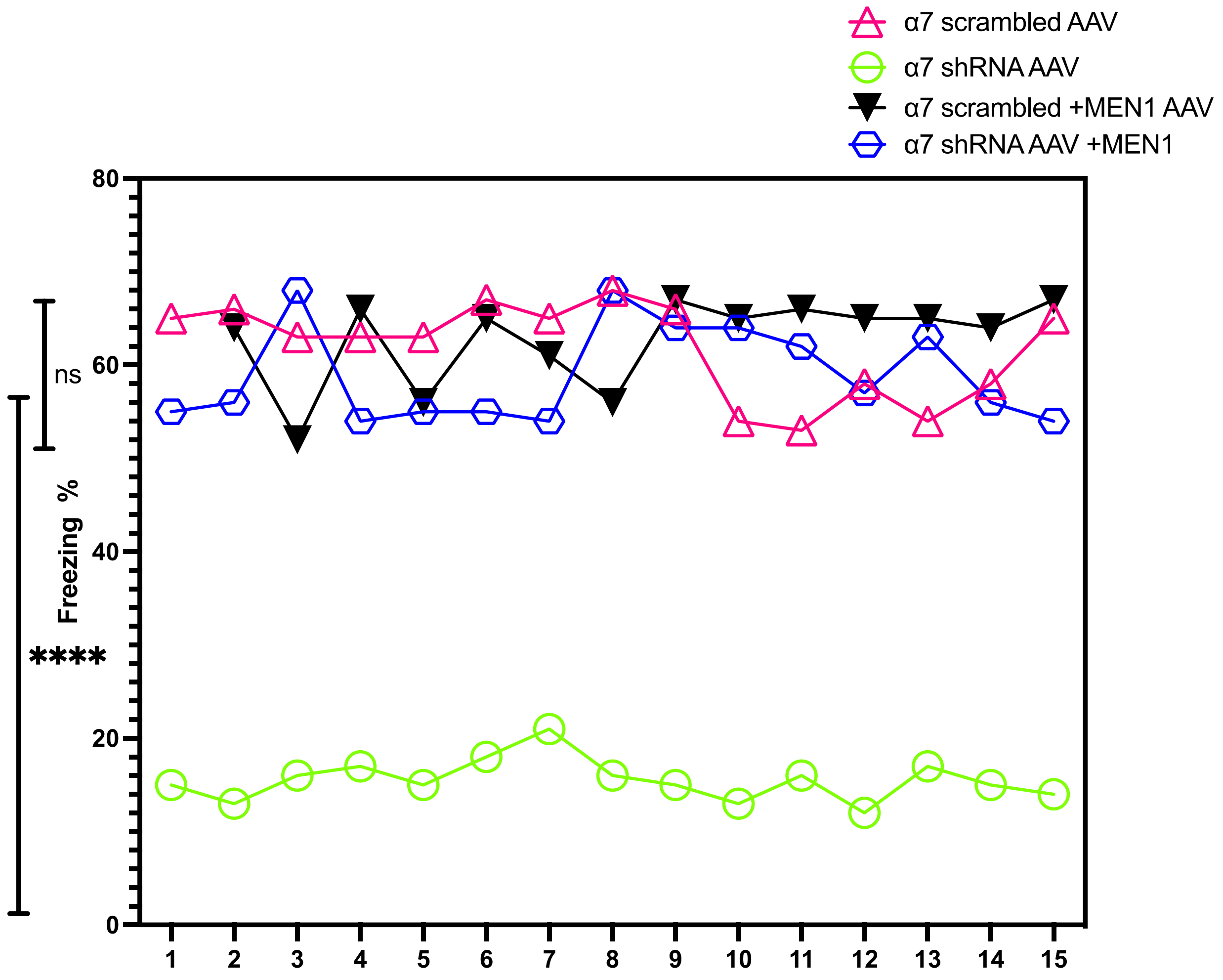

FIGURE S6

(A) Summary data, freezing percentage in four groups (n=15). Data shows deficits in learning and memory in α7 KD mice, whereas improvement in learning and memory in α7 KD+ MEN1 AAV mice. Scale bar, (Ai-iv) 20 µm, (Aiv-vi) 25 µm.
